# Supplementary material for: Causal interplay between lactose intolerance and gut microbiota: a combined bidirectional Mendelian randomization and in vivo validation study
Source: Front Nutr. 2026 Jun 1;13:1803337. doi: 10.3389/fnut.2026.1803337 (PMC13265576; doi:10.3389/fnut.2026.1803337)
Supplement: Supplementary file 1 [file Data_Sheet_1.zip › supplementary materials/Forward/forest plot/ebi-a-GCST90027755.finngen_R12_E4_LACTONAS.pdf]

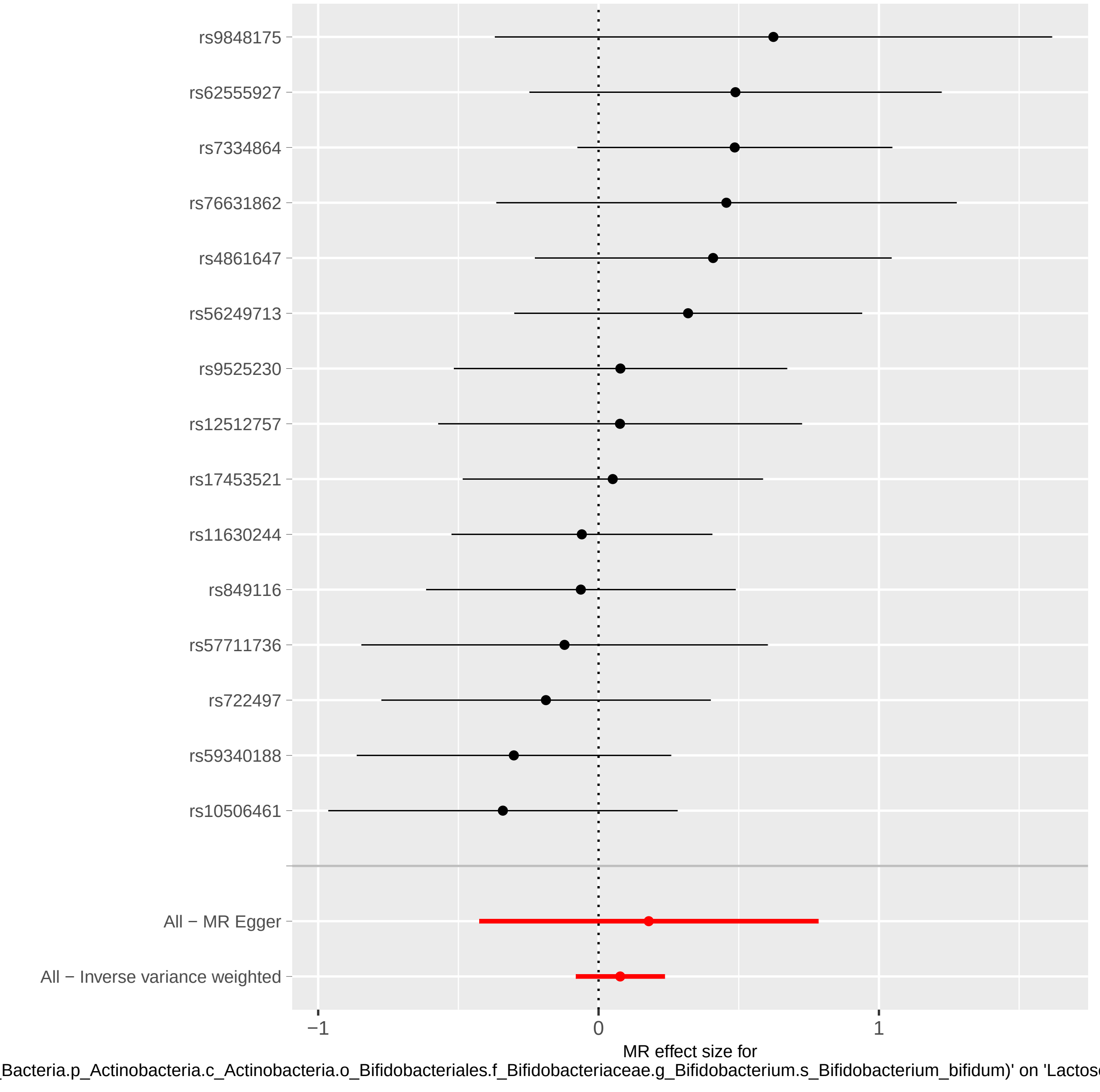
rs9848175
rs62555927
rs7334864
rs76631862
rs4861647
rs56249713
rs9525230
rs12512757
rs17453521
rs11630244
rs849116
rs57711736
rs722497
rs59340188
rs10506461
All − MR Egger
All − Inverse variance weighted
−1
0
1
MR effect size for
_Bacteria.p_Actinobacteria.c_Actinobacteria.o_Bifidobacteriales.f_Bifidobacteriaceae.g_Bifidobacterium.s_Bifidobacterium_bifidum)' on 'Lactos
